# Diacetato­(*N*,*N*-diethyl­ethylenediamine)­zinc(II)

**DOI:** 10.1107/S1600536810027418

**Published:** 2010-07-17

**Authors:** Young-Inn Kim, Sun-Young Yun, Taewoo Lee, Sung Kwon Kang

**Affiliations:** a Department of Chemistry Education, Interdisciplinary Program of Advanced Information and Display Materials and Center for Plastic Information Systems, Pusan National University, Busan 609-735, Republic of Korea; b Department of Chemistry, Chungnam National University, Daejeon 305-764, Republic of Korea

## Abstract

In the title compound, [Zn(CH_3_COO)_2_(C_6_H_16_N_2_)], the Zn^II^ atom is coordinated by two N atoms of one bidentate diethyl­ethylenediamine ligand and two O atoms of two acetate anions in a distorted tetra­hedral geometry. The acetate ligands are asymmetrically coordinated to the Zn atom with two different C—O distances of 1.234 (4) and 1.275 (4) Å. The dihedral angle between the N/Zn/N and O/Zn/O planes is 83.11 (8)°. There are two independent mol­ecules in the asymmetric unit. N—H⋯O hydrogen bonding links mol­ecules into a three-dimensional network.

## Related literature

For general background to luminescent compounds, see: Xu *et al.* (2008 ▶); Son *et al.* (2008 ▶). For the synthesis and structures of Zn^II^ metal complexes, see: Kim *et al.* (2007*a* ▶,*b* ▶); Seo *et al.* (2009 ▶); Das *et al.* (2006 ▶).
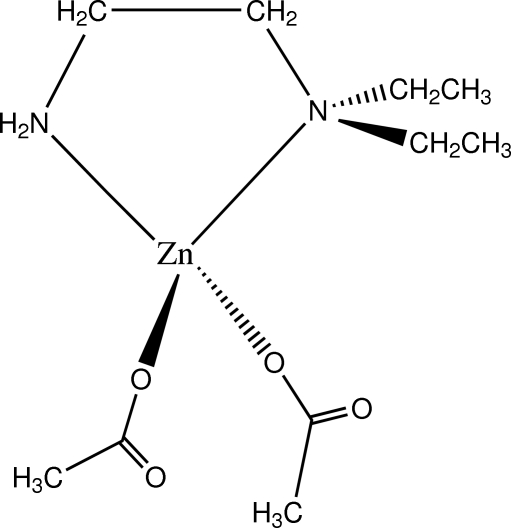


## Experimental

### 

#### Crystal data

[Zn(C_2_H_3_O_2_)_2_(C_6_H_16_N_2_)]
*M* *_r_* = 299.67
Monoclinic, 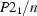

*a* = 7.5495 (1) Å
*b* = 13.3244 (2) Å
*c* = 27.5543 (4) Å
β = 94.617 (1)°
*V* = 2762.76 (7) Å^3^
*Z* = 8
Mo *K*α radiation
μ = 1.78 mm^−1^
*T* = 173 K
0.18 × 0.10 × 0.1 mm

#### Data collection

Bruker SMART CCD area-detector diffractometer
Absorption correction: multi-scan (*SADABS*; Bruker, 2002 ▶) *T*_min_ = 0.722, *T*_max_ = 0.834
27467 measured reflections
6837 independent reflections
5523 reflections with *I* > 2σ(*I*)
*R*_int_ = 0.033

#### Refinement

*R*[*F* ^2^ > 2σ(*F* ^2^)] = 0.047
*wR*(*F* ^2^) = 0.126
*S* = 1.04
6837 reflections
309 parameters
H-atom parameters constrained
Δρ_max_ = 1.74 e Å^−3^
Δρ_min_ = −0.95 e Å^−3^

### 

Data collection: *SMART* (Bruker, 2002 ▶); cell refinement: *SAINT* (Bruker, 2002 ▶); data reduction: *SAINT*; program(s) used to solve structure: *SHELXS97* (Sheldrick, 2008 ▶); program(s) used to refine structure: *SHELXL97* (Sheldrick, 2008 ▶); molecular graphics: *ORTEP-3 for Windows* (Farrugia, 1997 ▶); software used to prepare material for publication: *WinGX* (Farrugia, 1999 ▶).

## Supplementary Material

Crystal structure: contains datablocks global, I. DOI: 10.1107/S1600536810027418/jh2180sup1.cif

Structure factors: contains datablocks I. DOI: 10.1107/S1600536810027418/jh2180Isup2.hkl

Additional supplementary materials:  crystallographic information; 3D view; checkCIF report

## Figures and Tables

**Table 1:** Hydrogen-bond geometry (Å, °)

*D*—H⋯*A*	*D*—H	H⋯*A*	*D*⋯*A*	*D*—H⋯*A*
N4—H4*A*⋯O27^i^	0.9	2.02	2.904 (3)	168
N4—H4*B*⋯O31^ii^	0.9	2.16	3.032 (4)	163
N20—H20*A*⋯O15^iii^	0.9	2.01	2.911 (4)	176
N20—H20*B*⋯O11^iv^	0.9	2.06	2.925 (4)	160

Symmetry codes: (i) 
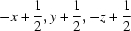
; (ii) 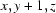; (iii) 
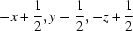
; (iv) 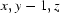.

**Fig. 1.**
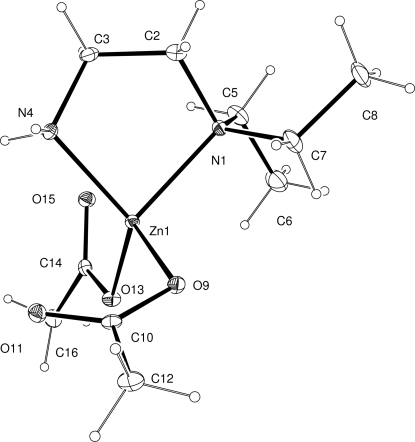
Molecular structure of (l), showing the atom-numbering scheme and 30% probability ellipsoids.

## supplementary crystallographic information

### Comment

Luminescent metal coordination compounds have attracted considerable attention for their potential applications in electroluminescent displays (Xu, *et al.* 2008). Among many coordination compounds, Zn^II^ metal complexes are of great interest due to their facile synthesis, tunable emission color and good electroluminescent (EL) properties (Son, *et al.* 2008). Recently, we reported blue fluorescent zinc(II) complexes with nitrogen-containing ligand to develop luminescent materials (Seo, *et al.* 2009; Kim *et al.*,  2007*a*; Kim *et al.*, 2007*b*). In an extension of this study, here we prepared novel zinc(II) complex with *N*,*N*-diethylethylenediamine and structurally studied. In the title compound, the Zn^II^ atom is coordinated by two N atoms of one bidentate diethylethylenediamine ligand and two O atoms of two acetate anions in a distorted tetrahedral geometry. The acetate ligands are asymmetrically coordinated to Zn atom with two different C—O distances of 1.234 (4) and 1.275 (4) Å. The dihedral angle between the N1/Zn1/N4 plane and O9/Zn1/O13 plane is 83.11 (8) °. N—H···O hydrogen bonding links molecules into a three-dimensional network.

The title compound shows an intense deep-blue emission at 402 nm attributed to ^1^(π - π^*^) intraligand charge transfer(ILCT) transition in CHCl_3_ upon 300 nm excitation and exhibits increased quantum yield of 5.47% compared with that of free ligand of 0.45%. The chelation of the ligand to Zn^II^ increased the rigidity of the ligand and thus reduced the loss of energy by thermal vibrational decay, resulting in enhancing the quantum yield in the title coordination compound (Das, *et al.* 2006).

### Experimental

A solution of zinc acetate (2.195 g, 10.0 mmol) and *N*,*N*-diethylethylenediamine (1.14 g, 10.0 mmol) in absolute ethanol (50 ml) was stirred for 8 h at room temperature under a nitrogen atmosphere. The resulting colourless solution was allowed to stand at room temperature for two weeks to produce colorless crystals (yield 65.0%) suitable for X-ray diffraction. Anal. calcd. for C_10_H_22_N_2_O_4_Zn: C, 40.08; H, 7.40; N, 8.57. Found: C, 38.69; H, 7.18; N, 8.57.

### Refinement

All H atoms were positioned geometrically and refined using a riding model, with N—H = 0.90 Å, and with *U*_iso_(H) = 1.2*U*_eq_(N) for NH_2_, C—H = 0.96 - 0.97 Å, and with *U*_iso_(H) = 1.2*U*_eq_(C) for methylene and 1.5*U*_eq_(C) for methyl H atoms.

### Crystal data

**Table:**

[Zn(C_2_H_3_O_2_)_2_(C_6_H_16_N_2_)]	*F*(000) = 1264
*M**_r_* = 299.67	*D*_x_ = 1.441 Mg m^−^^3^
Monoclinic, *P*2_1_/*n*	Mo *K*α radiation, λ = 0.71073 Å
Hall symbol: -P 2yn	Cell parameters from 8610 reflections
*a* = 7.5495 (1) Å	θ = 2.8–28.0°
*b* = 13.3244 (2) Å	µ = 1.78 mm^−^^1^
*c* = 27.5543 (4) Å	*T* = 173 K
β = 94.617 (1)°	Block, colourless
*V* = 2762.76 (7) Å^3^	0.18 × 0.1 × 0.1 mm
*Z* = 8	

### Data collection

**Table:**

Bruker SMART CCD area-detector diffractometer	5523 reflections with *I* > 2σ(*I*)
φ and ω scans	*R*_int_ = 0.033
Absorption correction: multi-scan (*SADABS*; Bruker, 2002)	θ_max_ = 28.3°, θ_min_ = 1.5°
*T*_min_ = 0.722, *T*_max_ = 0.834	*h* = −10→10
27467 measured reflections	*k* = −17→17
6837 independent reflections	*l* = −35→36

### Refinement

**Table:**

Refinement on *F*^2^	0 restraints
Least-squares matrix: full	H-atom parameters constrained
*R*[*F*^2^ > 2σ(*F*^2^)] = 0.047	*w* = 1/[σ^2^(*F*_o_^2^) + (0.059*P*)^2^ + 4.8891*P*] where *P* = (*F*_o_^2^ + 2*F*_c_^2^)/3
*wR*(*F*^2^) = 0.126	(Δ/σ)_max_ = 0.001
*S* = 1.04	Δρ_max_ = 1.74 e Å^−^^3^
6837 reflections	Δρ_min_ = −0.95 e Å^−^^3^
309 parameters	

### Special details

**Table:**

Geometry. All e.s.d.'s (except the e.s.d. in the dihedral angle between two l.s. planes) are estimated using the full covariance matrix. The cell e.s.d.'s are taken into account individually in the estimation of e.s.d.'s in distances, angles and torsion angles; correlations between e.s.d.'s in cell parameters are only used when they are defined by crystal symmetry. An approximate (isotropic) treatment of cell e.s.d.'s is used for estimating e.s.d.'s involving l.s. planes.

### Fractional atomic coordinates and isotropic or equivalent isotropic displacement parameters (Å^2^)

**Table:**

	*x*	*y*	*z*	*U*_iso_*/*U*_eq_	
Zn1	0.16918 (4)	0.56462 (3)	0.137611 (12)	0.02318 (10)	
N1	−0.0915 (4)	0.5126 (2)	0.11436 (10)	0.0288 (6)	
C2	−0.2078 (5)	0.5939 (4)	0.13401 (16)	0.0501 (11)	
H2A	−0.2184	0.6491	0.111	0.06*	
H2B	−0.3259	0.567	0.137	0.06*	
C3	−0.1366 (5)	0.6315 (3)	0.18130 (15)	0.0401 (9)	
H3A	−0.1437	0.5792	0.2056	0.048*	
H3B	−0.2078	0.6878	0.1907	0.048*	
N4	0.0500 (3)	0.6638 (2)	0.18003 (9)	0.0263 (6)	
H4A	0.1047	0.6642	0.2103	0.032*	
H4B	0.055	0.7261	0.1675	0.032*	
C5	−0.1389 (5)	0.4146 (3)	0.13401 (18)	0.0493 (10)	
H5A	−0.1216	0.4165	0.1693	0.059*	
H5B	−0.2636	0.4013	0.1251	0.059*	
C6	−0.0318 (7)	0.3335 (4)	0.1158 (2)	0.0727 (16)	
H6A	−0.067	0.2707	0.1292	0.109*	
H6B	0.0916	0.3455	0.1253	0.109*	
H6C	−0.0495	0.3309	0.0809	0.109*	
C7	−0.1186 (6)	0.5195 (4)	0.06090 (15)	0.0519 (11)	
H7A	−0.032	0.4767	0.047	0.062*	
H7B	−0.0946	0.588	0.0514	0.062*	
C8	−0.3044 (6)	0.4902 (5)	0.03852 (18)	0.0662 (15)	
H8A	−0.3085	0.4966	0.0037	0.099*	
H8B	−0.3915	0.5338	0.0509	0.099*	
H8C	−0.3293	0.4221	0.0469	0.099*	
O9	0.2504 (3)	0.62175 (18)	0.07728 (8)	0.0315 (5)	
C10	0.3632 (4)	0.6901 (3)	0.08970 (13)	0.0295 (7)	
O11	0.4063 (3)	0.71228 (19)	0.13251 (10)	0.0374 (6)	
C12	0.4416 (5)	0.7456 (3)	0.04874 (16)	0.0451 (10)	
H12A	0.3834	0.8093	0.044	0.068*	
H12B	0.4249	0.7068	0.0194	0.068*	
H12C	0.5663	0.756	0.0568	0.068*	
O13	0.3570 (3)	0.47619 (19)	0.16413 (9)	0.0331 (5)	
C14	0.3221 (4)	0.4350 (2)	0.20440 (12)	0.0281 (7)	
O15	0.1721 (3)	0.4354 (2)	0.21913 (9)	0.0367 (6)	
C16	0.4745 (5)	0.3854 (3)	0.23375 (14)	0.0402 (9)	
H16A	0.4735	0.4049	0.2673	0.06*	
H16B	0.5845	0.4059	0.2216	0.06*	
H16C	0.4627	0.3138	0.2311	0.06*	
Zn2	0.26193 (4)	0.00571 (3)	0.136874 (12)	0.02231 (10)	
N17	0.5069 (4)	0.0707 (2)	0.12031 (10)	0.0323 (6)	
C18	0.6250 (5)	0.0412 (3)	0.16251 (16)	0.0449 (8)	
H18A	0.6034	0.0843	0.1898	0.054*	
H18B	0.7473	0.0508	0.1551	0.054*	
C19	0.5986 (5)	−0.0656 (4)	0.17656 (18)	0.0527 (11)	
H19A	0.6471	−0.109	0.1527	0.063*	
H19B	0.6637	−0.0782	0.2078	0.063*	
N20	0.4078 (3)	−0.0914 (2)	0.18006 (10)	0.0281 (6)	
H20A	0.3795	−0.0858	0.2111	0.034*	
H20B	0.3865	−0.1549	0.1701	0.034*	
C21	0.4946 (6)	0.1876 (4)	0.12071 (18)	0.0593 (13)	
H21A	0.4538	0.2092	0.1515	0.071*	
H21B	0.6122	0.2154	0.1182	0.071*	
C22	0.3713 (8)	0.2277 (4)	0.0800 (2)	0.0807 (19)	
H22A	0.3689	0.2997	0.0817	0.121*	
H22B	0.2538	0.2019	0.0829	0.121*	
H22C	0.4119	0.2073	0.0494	0.121*	
C23	0.5562 (5)	0.0365 (3)	0.07219 (14)	0.0371 (8)	
H23A	0.461	0.0547	0.0481	0.045*	
H23B	0.5635	−0.0362	0.0727	0.045*	
C24	0.7298 (5)	0.0772 (3)	0.05540 (15)	0.0408 (9)	
H24A	0.7485	0.0503	0.0239	0.061*	
H24B	0.8266	0.058	0.0782	0.061*	
H24C	0.7238	0.1491	0.0535	0.061*	
O25	0.0876 (3)	0.08852 (18)	0.16648 (8)	0.0311 (5)	
C26	0.1454 (4)	0.1347 (2)	0.20555 (11)	0.0263 (6)	
O27	0.3035 (3)	0.1405 (2)	0.21990 (8)	0.0350 (6)	
C28	0.0083 (5)	0.1842 (3)	0.23464 (15)	0.0424 (9)	
H28A	0.0292	0.1656	0.2683	0.064*	
H28B	−0.1083	0.1627	0.2225	0.064*	
H28C	0.017	0.2558	0.2316	0.064*	
O29	0.1591 (3)	−0.04624 (18)	0.07437 (8)	0.0291 (5)	
C30	0.0457 (4)	−0.1155 (3)	0.08104 (12)	0.0265 (6)	
O31	0.0095 (3)	−0.1432 (2)	0.12188 (9)	0.0390 (6)	
C32	−0.0414 (5)	−0.1633 (3)	0.03550 (14)	0.0404 (9)	
H32A	0.0242	−0.2221	0.0277	0.061*	
H32B	−0.0426	−0.1164	0.009	0.061*	
H32C	−0.1611	−0.1819	0.0409	0.061*	

### Atomic displacement parameters (Å^2^)

**Table:**

	*U*^11^	*U*^22^	*U*^33^	*U*^12^	*U*^13^	*U*^23^
Zn1	0.02171 (17)	0.0258 (2)	0.02222 (18)	−0.00040 (14)	0.00278 (12)	0.00037 (14)
N1	0.0260 (13)	0.0326 (16)	0.0278 (13)	−0.0040 (11)	0.0014 (10)	−0.0028 (12)
C2	0.0320 (18)	0.061 (3)	0.057 (3)	0.0071 (19)	0.0044 (17)	−0.010 (2)
C3	0.0305 (17)	0.042 (2)	0.050 (2)	0.0040 (16)	0.0134 (15)	−0.0109 (18)
N4	0.0319 (13)	0.0245 (14)	0.0229 (12)	0.0010 (11)	0.0041 (10)	0.0007 (11)
C5	0.038 (2)	0.042 (2)	0.066 (3)	−0.0063 (18)	−0.0084 (19)	0.008 (2)
C6	0.052 (3)	0.066 (4)	0.098 (4)	−0.007 (2)	−0.007 (3)	−0.018 (3)
C7	0.041 (2)	0.073 (3)	0.039 (2)	−0.015 (2)	−0.0125 (17)	0.010 (2)
C8	0.047 (2)	0.093 (4)	0.054 (3)	−0.011 (3)	−0.023 (2)	−0.003 (3)
O9	0.0339 (12)	0.0335 (13)	0.0282 (11)	−0.0064 (10)	0.0091 (9)	0.0018 (10)
C10	0.0259 (15)	0.0251 (17)	0.0385 (18)	0.0060 (13)	0.0090 (13)	0.0050 (14)
O11	0.0331 (12)	0.0341 (14)	0.0445 (15)	0.0012 (11)	−0.0006 (10)	−0.0050 (11)
C12	0.045 (2)	0.038 (2)	0.056 (2)	−0.0040 (17)	0.0204 (18)	0.0119 (19)
O13	0.0303 (11)	0.0348 (14)	0.0346 (13)	0.0048 (10)	0.0050 (10)	0.0088 (11)
C14	0.0330 (16)	0.0201 (16)	0.0303 (16)	−0.0008 (13)	−0.0024 (13)	0.0002 (13)
O15	0.0337 (12)	0.0468 (16)	0.0300 (12)	0.0006 (11)	0.0049 (10)	0.0037 (11)
C16	0.0408 (19)	0.038 (2)	0.041 (2)	0.0070 (16)	−0.0059 (15)	0.0058 (17)
Zn2	0.02020 (17)	0.0247 (2)	0.02207 (18)	−0.00164 (13)	0.00178 (12)	−0.00151 (14)
N17	0.0293 (13)	0.0405 (17)	0.0274 (14)	−0.0142 (13)	0.0038 (11)	−0.0009 (12)
C18	0.0407 (19)	0.04	0.054 (2)	−0.0093 (17)	0.0030 (18)	−0.0051 (19)
C19	0.0283 (18)	0.060 (3)	0.067 (3)	0.0067 (18)	−0.0128 (18)	0.011 (2)
N20	0.0304 (13)	0.0291 (15)	0.0246 (13)	0.0020 (11)	0.0014 (10)	0.0001 (11)
C21	0.04	0.076 (3)	0.066 (3)	−0.033 (2)	0.0304 (19)	−0.026 (3)
C22	0.079 (4)	0.054 (3)	0.116 (5)	0.017 (3)	0.053 (4)	0.020 (3)
C23	0.0349 (17)	0.038 (2)	0.0410 (19)	−0.0088 (15)	0.0162 (15)	−0.0122 (16)
C24	0.0322 (17)	0.046 (2)	0.046 (2)	0.0000 (16)	0.0165 (15)	−0.0023 (18)
O25	0.0262 (11)	0.0336 (13)	0.0335 (12)	0.0022 (10)	0.0019 (9)	−0.0097 (10)
C26	0.0290 (15)	0.0232 (16)	0.0271 (15)	−0.0001 (13)	0.0048 (12)	0.0016 (12)
O27	0.0296 (12)	0.0475 (16)	0.0278 (11)	−0.0012 (11)	0.0015 (9)	−0.0052 (11)
C28	0.0360 (18)	0.049 (2)	0.043 (2)	0.0029 (17)	0.0077 (16)	−0.0154 (18)
O29	0.0261 (11)	0.0342 (13)	0.0267 (11)	−0.0065 (10)	0.0008 (9)	−0.0048 (10)
C30	0.0211 (13)	0.0266 (17)	0.0319 (16)	0.0024 (12)	0.0030 (12)	−0.0031 (13)
O31	0.0446 (14)	0.0352 (14)	0.0381 (14)	0.0001 (12)	0.0085 (11)	0.0066 (11)
C32	0.0325 (17)	0.043 (2)	0.045 (2)	−0.0111 (16)	0.0002 (15)	−0.0105 (17)

### Geometric parameters (Å, °)

**Table:**

Zn1—O13	1.941 (2)	Zn2—O25	1.946 (2)
Zn1—O9	1.971 (2)	Zn2—O29	1.958 (2)
Zn1—N4	2.023 (3)	Zn2—N20	2.023 (3)
Zn1—N1	2.136 (3)	Zn2—N17	2.124 (3)
N1—C5	1.468 (5)	N17—C18	1.461 (5)
N1—C7	1.474 (5)	N17—C23	1.478 (4)
N1—C2	1.522 (5)	N17—C21	1.560 (6)
C2—C3	1.458 (6)	C18—C19	1.493 (6)
C2—H2A	0.97	C18—H18A	0.97
C2—H2B	0.97	C18—H18B	0.97
C3—N4	1.476 (4)	C19—N20	1.491 (5)
C3—H3A	0.97	C19—H19A	0.97
C3—H3B	0.97	C19—H19B	0.97
N4—H4A	0.9	N20—H20A	0.9
N4—H4B	0.9	N20—H20B	0.9
C5—C6	1.464 (7)	C21—C22	1.497 (8)
C5—H5A	0.97	C21—H21A	0.97
C5—H5B	0.97	C21—H21B	0.97
C6—H6A	0.96	C22—H22A	0.96
C6—H6B	0.96	C22—H22B	0.96
C6—H6C	0.96	C22—H22C	0.96
C7—C8	1.536 (5)	C23—C24	1.524 (5)
C7—H7A	0.97	C23—H23A	0.97
C7—H7B	0.97	C23—H23B	0.97
C8—H8A	0.96	C24—H24A	0.96
C8—H8B	0.96	C24—H24B	0.96
C8—H8C	0.96	C24—H24C	0.96
O9—C10	1.275 (4)	O25—C26	1.285 (4)
C10—O11	1.234 (4)	C26—O27	1.229 (4)
C10—C12	1.509 (5)	C26—C28	1.510 (5)
C12—H12A	0.96	C28—H28A	0.96
C12—H12B	0.96	C28—H28B	0.96
C12—H12C	0.96	C28—H28C	0.96
O13—C14	1.284 (4)	O29—C30	1.282 (4)
C14—O15	1.233 (4)	C30—O31	1.236 (4)
C14—C16	1.505 (5)	C30—C32	1.510 (5)
C16—H16A	0.96	C32—H32A	0.96
C16—H16B	0.96	C32—H32B	0.96
C16—H16C	0.96	C32—H32C	0.96
			
O13—Zn1—O9	106.53 (10)	O25—Zn2—O29	109.61 (10)
O13—Zn1—N4	121.65 (11)	O25—Zn2—N20	118.07 (11)
O9—Zn1—N4	114.72 (11)	O29—Zn2—N20	116.73 (11)
O13—Zn1—N1	122.93 (11)	O25—Zn2—N17	119.27 (11)
O9—Zn1—N1	102.37 (10)	O29—Zn2—N17	104.20 (10)
N4—Zn1—N1	86.76 (11)	N20—Zn2—N17	86.84 (11)
C5—N1—C7	114.0 (3)	C18—N17—C23	116.7 (3)
C5—N1—C2	109.7 (3)	C18—N17—C21	107.2 (3)
C7—N1—C2	105.9 (3)	C23—N17—C21	109.5 (3)
C5—N1—Zn1	115.1 (2)	C18—N17—Zn2	101.9 (2)
C7—N1—Zn1	109.3 (2)	C23—N17—Zn2	110.7 (2)
C2—N1—Zn1	101.9 (2)	C21—N17—Zn2	110.6 (2)
C3—C2—N1	112.4 (3)	N17—C18—C19	112.1 (3)
C3—C2—H2A	109.1	N17—C18—H18A	109.2
N1—C2—H2A	109.1	C19—C18—H18A	109.2
C3—C2—H2B	109.1	N17—C18—H18B	109.2
N1—C2—H2B	109.1	C19—C18—H18B	109.2
H2A—C2—H2B	107.9	H18A—C18—H18B	107.9
C2—C3—N4	111.3 (3)	N20—C19—C18	112.7 (3)
C2—C3—H3A	109.4	N20—C19—H19A	109
N4—C3—H3A	109.4	C18—C19—H19A	109
C2—C3—H3B	109.4	N20—C19—H19B	109
N4—C3—H3B	109.4	C18—C19—H19B	109
H3A—C3—H3B	108	H19A—C19—H19B	107.8
C3—N4—Zn1	107.0 (2)	C19—N20—Zn2	107.3 (2)
C3—N4—H4A	110.3	C19—N20—H20A	110.3
Zn1—N4—H4A	110.3	Zn2—N20—H20A	110.3
C3—N4—H4B	110.3	C19—N20—H20B	110.3
Zn1—N4—H4B	110.3	Zn2—N20—H20B	110.3
H4A—N4—H4B	108.6	H20A—N20—H20B	108.5
C6—C5—N1	111.9 (4)	C22—C21—N17	112.6 (4)
C6—C5—H5A	109.2	C22—C21—H21A	109.1
N1—C5—H5A	109.2	N17—C21—H21A	109.1
C6—C5—H5B	109.2	C22—C21—H21B	109.1
N1—C5—H5B	109.2	N17—C21—H21B	109.1
H5A—C5—H5B	107.9	H21A—C21—H21B	107.8
C5—C6—H6A	109.5	C21—C22—H22A	109.5
C5—C6—H6B	109.5	C21—C22—H22B	109.5
H6A—C6—H6B	109.5	H22A—C22—H22B	109.5
C5—C6—H6C	109.5	C21—C22—H22C	109.5
H6A—C6—H6C	109.5	H22A—C22—H22C	109.5
H6B—C6—H6C	109.5	H22B—C22—H22C	109.5
N1—C7—C8	115.7 (4)	N17—C23—C24	116.6 (3)
N1—C7—H7A	108.3	N17—C23—H23A	108.1
C8—C7—H7A	108.3	C24—C23—H23A	108.1
N1—C7—H7B	108.3	N17—C23—H23B	108.1
C8—C7—H7B	108.3	C24—C23—H23B	108.1
H7A—C7—H7B	107.4	H23A—C23—H23B	107.3
C7—C8—H8A	109.5	C23—C24—H24A	109.5
C7—C8—H8B	109.5	C23—C24—H24B	109.5
H8A—C8—H8B	109.5	H24A—C24—H24B	109.5
C7—C8—H8C	109.5	C23—C24—H24C	109.5
H8A—C8—H8C	109.5	H24A—C24—H24C	109.5
H8B—C8—H8C	109.5	H24B—C24—H24C	109.5
C10—O9—Zn1	107.2 (2)	C26—O25—Zn2	115.3 (2)
O11—C10—O9	123.1 (3)	O27—C26—O25	123.7 (3)
O11—C10—C12	120.6 (3)	O27—C26—C28	119.4 (3)
O9—C10—C12	116.3 (3)	O25—C26—C28	116.9 (3)
C10—C12—H12A	109.5	C26—C28—H28A	109.5
C10—C12—H12B	109.5	C26—C28—H28B	109.5
H12A—C12—H12B	109.5	H28A—C28—H28B	109.5
C10—C12—H12C	109.5	C26—C28—H28C	109.5
H12A—C12—H12C	109.5	H28A—C28—H28C	109.5
H12B—C12—H12C	109.5	H28B—C28—H28C	109.5
C14—O13—Zn1	112.9 (2)	C30—O29—Zn2	110.5 (2)
O15—C14—O13	122.7 (3)	O31—C30—O29	123.0 (3)
O15—C14—C16	120.5 (3)	O31—C30—C32	121.1 (3)
O13—C14—C16	116.8 (3)	O29—C30—C32	115.8 (3)
C14—C16—H16A	109.5	C30—C32—H32A	109.5
C14—C16—H16B	109.5	C30—C32—H32B	109.5
H16A—C16—H16B	109.5	H32A—C32—H32B	109.5
C14—C16—H16C	109.5	C30—C32—H32C	109.5
H16A—C16—H16C	109.5	H32A—C32—H32C	109.5
H16B—C16—H16C	109.5	H32B—C32—H32C	109.5

### Hydrogen-bond geometry (Å, °)

**Table:**

*D*—H···*A*	*D*—H	H···*A*	*D*···*A*	*D*—H···*A*
N4—H4A···O27^i^	0.9	2.02	2.904 (3)	168
N4—H4B···O31^ii^	0.9	2.16	3.032 (4)	163
N20—H20A···O15^iii^	0.9	2.01	2.911 (4)	176
N20—H20B···O11^iv^	0.9	2.06	2.925 (4)	160

Symmetry codes: (i) −*x*+1/2, *y*+1/2, −*z*+1/2; (ii) *x*, *y*+1, *z*; (iii) −*x*+1/2, *y*−1/2, −*z*+1/2; (iv) *x*, *y*−1, *z*.
